# Tear Glucose Measurement by Reflectance Spectrum of a Nanoparticle Embedded Contact Lens

**DOI:** 10.1038/s41598-020-65103-z

**Published:** 2020-05-19

**Authors:** Sooyeon Kim, Hee-Jae Jeon, Sijin Park, Dong Yun Lee, Euiheon Chung

**Affiliations:** 1 Department of Biomedical Science and Engineering Physics and Photon Science, Gwangju Institute of Science and Technology (GIST), Gwangju, 61005 Republic of Korea; 2 Department of Bioengineering, College of Engineering, and BK21 PLUS Future Biopharmaceutical Human Resources Training and Research Team, Hanyang University, Seoul, 04763 Republic of Korea; 3 Institute of Nano Science & Technology (INST), Hanyang University, Seoul, 04763 Republic of Korea

**Keywords:** Health care, Optics and photonics, Biomedical engineering

## Abstract

Glucose level is a primary indicator in the diagnosis and treatment of diabetes mellitus. According to the correlation between glucose concentration in blood and tears, measuring tear glucose can be an alternative to traditional strips test for blood glucose. Thus, measuring tear glucose levels could provide noninvasive monitoring of blood glucose. As a biocompatible biosensor, a nanoparticle embedded contact lens (NECL) is developed which is composed of glucose oxidase and cerium oxide (III). Using spectroscopy, we found the detectable changes in reflection spectrum of contact lenses with respect to the glucose concentration, and developed correlation curve of the reflection spectrum with known glucose level. Furthermore, we assessed tear glucose level and compared blood glucose level with the diabetic mouse model to evaluate this approach. Our algorithm for regular monitoring of glucose using contact lens biosensor may lead to noninvasive monitoring of tear glucose level. NECL may provide simple and noninvasive glucose monitoring based on the spectral changes in contact lens biosensor.

## Introduction

Diabetes mellitus is one of the leading causes of death, and recently, the number of diabetic patients is rapidly increasing in developed countries^1^. Hyperglycemia potentially results in complications of the micro-vascular system that can cause cardiovascular disorders, nerve damage, and kidney failure. To prevent these multiple complications, regular blood glucose monitoring is necessary after the diagnosis of pre-diabetic or diabetic condition^2,3^.

Commercial ‘finger prick’ glucometer usually contains test strips and glucose oxidase or dehydrogenase to display the plasma glucose level from electrochemical analysis through the strip-based glucometer. Diabetes patients are required to monitor their blood glucose at least 4–5 times a day. However, this repeated pricking causes patients to feel discomfort^4^. Therefore, diverse types of glucose monitoring methods have been developed to accurately estimate blood glucose concentration using least invasive or non-invasive glucose sensors.

Compared to other biological fluids, such as saliva^5–7^ and sweat^8^, the volume of interstitial fluid (ISF)^9,10^ and tear^11^ are well maintained and their glucose concentration are relatively stable. For this reason, indirect blood glucose monitoring methods have been introduced by measuring tear glucose concentration along with the correlation between tear and blood glucose concentrations^12^. Tear glucose concentration analysis systems using enzyme-based amperometric and coulometric glucose sensors have been reported showing the correlation between tear and blood glucose concentration by collecting tear fluid into the glass capillary tube from rabbits or humans^13,14^. On the other hand, smart contact lenses integrated with a glucose sensor and analysis circuit have been developed to detect tear glucose while wearing^15,16^. To fabricate this sensor and circuit into a contact lens, micro-electro-mechanical system (MEMS) technique was applied to biosensor^17^ and wireless circuit and displays were recently demonstrated^15^.

Among diverse methods for indirect blood glucose monitoring, electrochemical^18–21^ and optical analysis^22–24^ have been investigated. Although the electrochemical analysis provides quantitative and continuous glucose monitoring by direct converting an electrical signal to the corresponding glucose concentration, this approach requires external power to connect with contact lens sensor with wire or wirelessly^11,25^. Additionally, multiple procedures for manufacturing contact lens sensors are expected to integrate the sensor and analysis circuit.

In terms of optical analysis methods, Elsherif *et al*., proposed a wearable contact lens optical sensor and compared a total of six groups at glucose concentrations ranging from 0 to 50 mM at every 10 mM interval^26^. Since the glucose concentration in tears is known to be in the range of 0.05–5 mM^27^, their demonstrated sensitivity was not practical enough. Another recent optical glucose sensor developed by an MIT group in 2020 provides higher sensitivity albeit requiring a high resolution optical spectrometer for distinguishing Raman signatures^28^. Their non-invasive glucose measurement using Raman spectroscopy requires a 830 nm-diode laser to measure the glucose concentration from the Raman shift. For this reason, the optical glucose sensor have to be more sensitive and simple.

**Figure 1:**
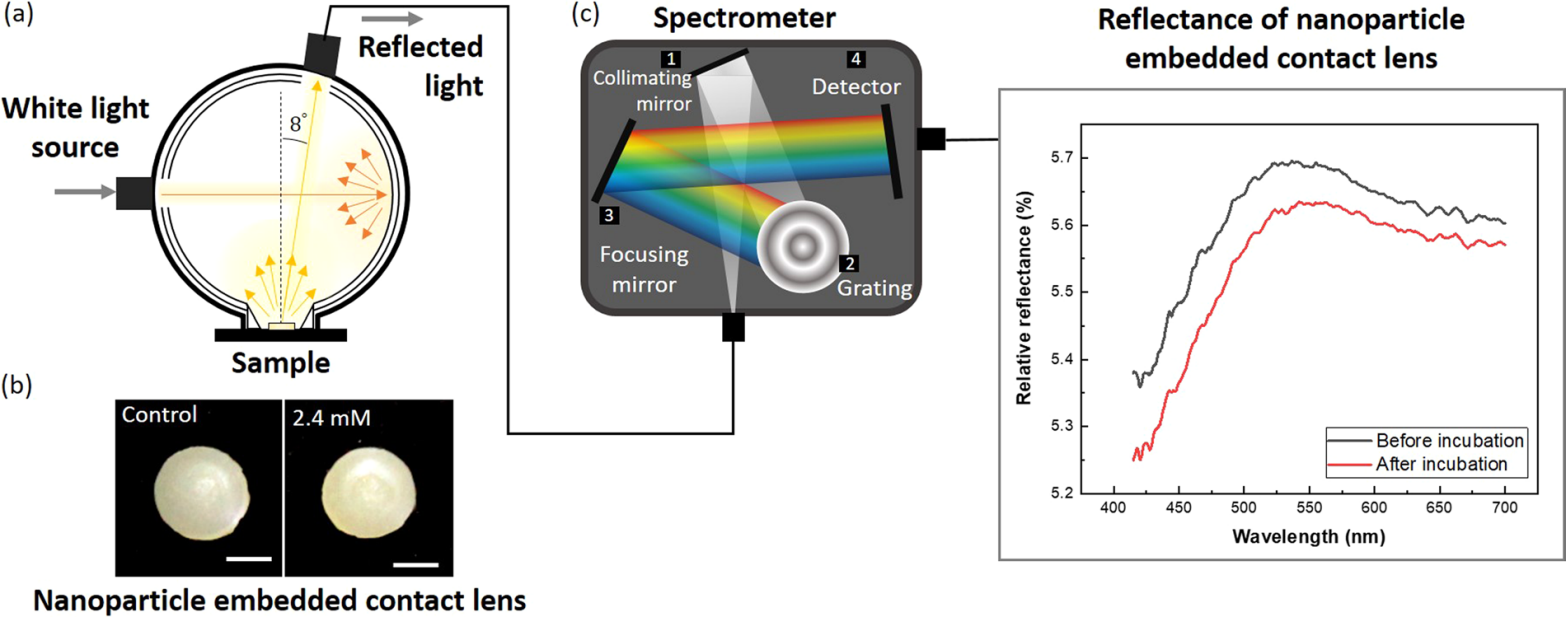
Schematic representation of the strategy for tear glucose detection by measuring the reflectance spectrum. (**a**) Configuration of the integrating sphere consisting of d/8° geometry. The external light source projects perpendicularly to the sample and the detection port were coupled with spectrometer 8-degree varied from the normal. (**b**) Photograph of NECL captured before reaction (left) and after reaction with 2.4 mM of glucose solution (right), mixed with artificial tear. Scale bar is 1 mm. (**c**) Collected light was quantitatively analyzed by this spectroscopic system. The tear glucose concentration was predicted by comparing the reflection spectra of control and reacted contact lens.

In this study, we introduce a novel spectroscopy-based measurement (Fig. 1) of tear glucose concentration with nanoparticle embedded contact lenses (NECL). After establishing a calibration curve to estimate the tear glucose level, it was compared with corresponding blood glucose level between non-diabetic and diabetic mouse groups.

## Results

### Spectrum collection

**Figure 2:**
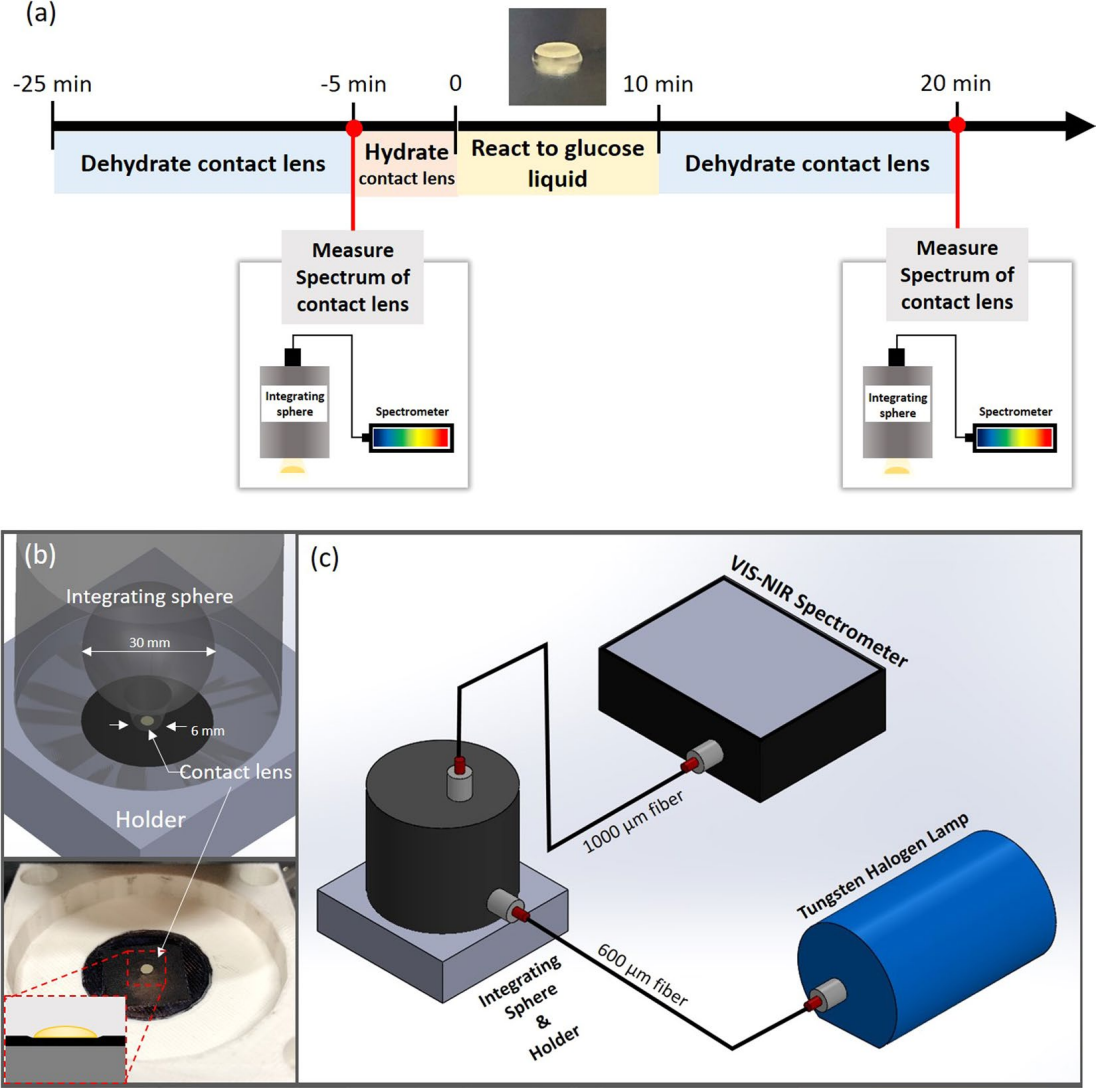
Schematic of experimental timeline Scheme of optical system for reflectance measurement. (**a**) Schematic of tear glucose sensing contact lens preparation, dehydration, measurement and study procedures in both calibration experiments. (**b**) Integrating sphere and the holder. The integrating sphere has a sphere with a diameter of 30 mm and 6 mm sample port. The tear glucose contact lens sensor is placed on the black plate. Photograph of the 3D printed holder and tear glucose contact lens sensor. (**c**) 3D structure of the reflectance spectrometer system. Tungsten halogen lamp is coupled with 600 µm fiber and spectrometer is coupled with 1000 µm fiber core diameter.

Spectroscopic measurements were carried out by a commercial spectrometer system working in the reflectance mode. The optical system consists of a spectrometer (FLAME-T-VIS-NIR-ES, Ocean Optics Inc., Dunedin, FL, USA), light source (HL-2000-HP-FHSA, Ocean Optics Inc., Dunedin, FL, USA) and integrating sphere (ISP-30-6-R, Ocean Optics Inc., Dunedin, FL, USA). The spectral data imported to the OceanView (Ocean Optics Inc., Dunedin, FL, USA). Additionally, we designed the holder to fix the integrating sphere and NECL position using Solidworks (SOLIDWORKS Corp., USA). Figure 2(c) shows the schematic diagram of the reflectance spectroscopic system. Scanning configurations for each sample includes an integration time of 100 ms and the signal was measured three times and averaged. Before every measurement the relative reflectance is calculated

1 $${\rm{Relative}}\,{\rm{reflectance}}=\frac{{R}_{s}-{R}_{b}}{{R}_{r}-{R}_{b}}$$

where, R_s_: Intensity of reflected light for sample; R_r_: Intensity of reflected light for the specular reflectance standard (STAN-SSL, Ocean Optics Inc., Dunedin, FL, USA); R_b_: Intensity of reflected light for the background.

Each NECL was placed on the 3D printed holder, under the integrating sphere port (Fig. 2(b)). The holder ensures the NECL place on the center of the plate and fix the integrating sphere. Inside the integrating sphere is coated PTFF, which has >95% reflectivity in the visible region. Out of 415–700 nm range was cut from each scanned spectra.

### Spectrum pre-treatment

The obtained spectral data were exported to MATLAB for data pre-treatment. The analytical information of reflectance spectra is often influenced by signal fluctuation and baseline drift, which are produced during the operational process. These influence factors will have adverse effects on the accuracy of the detection models. Therefore, before establishing the calibration model, the spectral data were first pretreated to reduce as much as interference information. These pretreatments were smoothing (SM), standard normal variate (SNV)^29^. The visualization was performed with third order polynomial fitting.

### Establishing calibration model

Tear solution was prepared with difference glucose concentrations of 0, 0.6, 1.2 and 2.4 mM, with artificial tear fluid (0.15% Sodium hyaluronate). All solutions were prepared on the same day of experiment. As depicted in Fig. 2(a), the NECL was dehydrated and measured before reacting to glucose liquid. A total of 25 NECLs were used to establish the calibration and 5 NECLs were used in each group. NECL was immersed in deionized water for 5 minutes for hydration. NECL was placed on a 5 µL drop of glucose solution. After reaction for 10 minutes, the remaining solution on the surface of NECL was removed with kimwipes and dehydrated for 20 minutes. All relative reflectance of NECL was measured after absolute dehydration under the room temperature.

**Figure 3:**
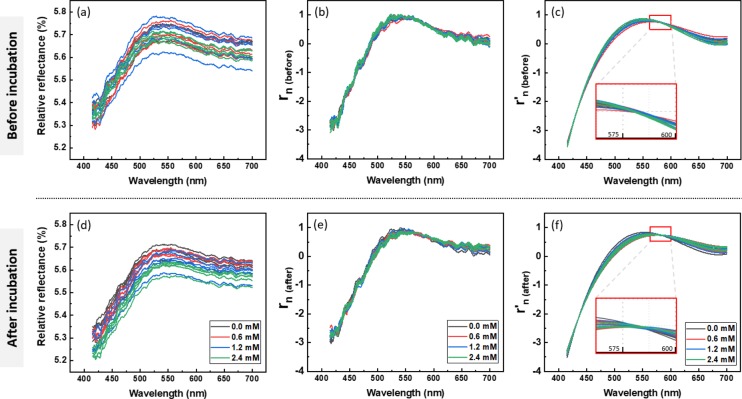
Spectrum analysis process for calibration. To remove the baseline difference in relative reflectance, SNV normalization was applied maintaining the original shape of contact lens reflectance. The normalized relative reflectance was 3^rd^ curve fitted and visualized. Relative reflectance of controls (**a**), reacted with glucose solution (**d**). Normalized relative reflectance of controls (**b**), reacted with glucose solution (**e**). 3^rd^ curve fitted and normalized relative reflectance (**c**), reacted with glucose solution (**f**).

The relative reflectance of the nanoparticle contact lenses in visible light was observed at less than 6% both before and after reaction condition. The relative reflectance of contact lenses was observed to have different baselines (Fig. 3(a,b)). SNV was applied to the relative reflectance to remove the baseline drift present throughout the spectrum. To remove noise due to low SNR, we fitted the relative reflectance to a 3rd order polynomial function. The tendency to decrease the relative reflectance at 2.4 mM group was maintained after the 3^rd^ curve fit (Fig. 3).

**Figure 4:**
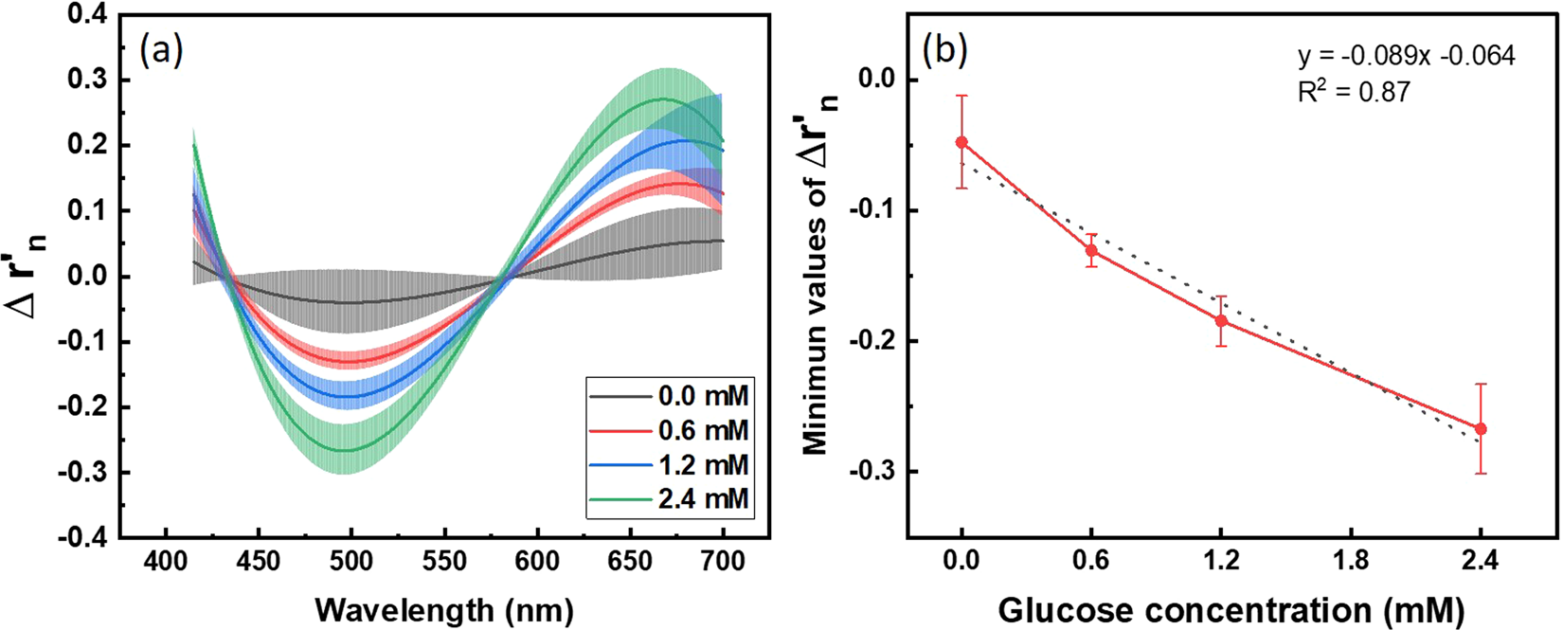
Result of calibration using 5 µL glucose solutions. (**a**) Difference of relative reflectance before and after reaction with 0.0 mM, 0.6 mM, 1.2 mM and 2.4 mM of glucose mixed in artificial tear. (**b**) Calibration curve. The linear curve fit based on all data points represents equation, y = −0.089x − 0.064 and R^2^ value was 0.87. Error bars indicate standard deviation (n = 5).

We measured the relative reflectance of each NECL before and after reaction and performed the one-to-one comparison. To visualize the change in the relative reflectance, the mean value of the difference value in relative reflectance were shown in Fig. 4(a). Error bars represent standard deviation. ∆r_n_ was defined as;

2 $${\Delta {\rm{r}}}_{{\rm{n}}}={{\rm{r}}}_{{\rm{n}}({\rm{after}})}-{{\rm{r}}}_{{\rm{n}}({\rm{before}})}$$

where, r_n_ is the normalized relative reflectance using SNV correction method. And r’_n_ is the 3^rd^ fitted relative reflectance.

In difference graph (Fig. 4(a)), point with the minimum values of all ${\Delta {\rm{r}}}_{{\rm{n}}}$ graphs were observed near 500 nm in common. As shown in Fig. 4(b), a linear relationship was obtained between the glucose concentration and changed relative reflectance of NECL (${R}^{2}$ > 0.87). Additionally, the differences between groups were all statistically significant (p < 0.01).

### Feasibility test *in vivo* animal experiment

**Figure 5:**
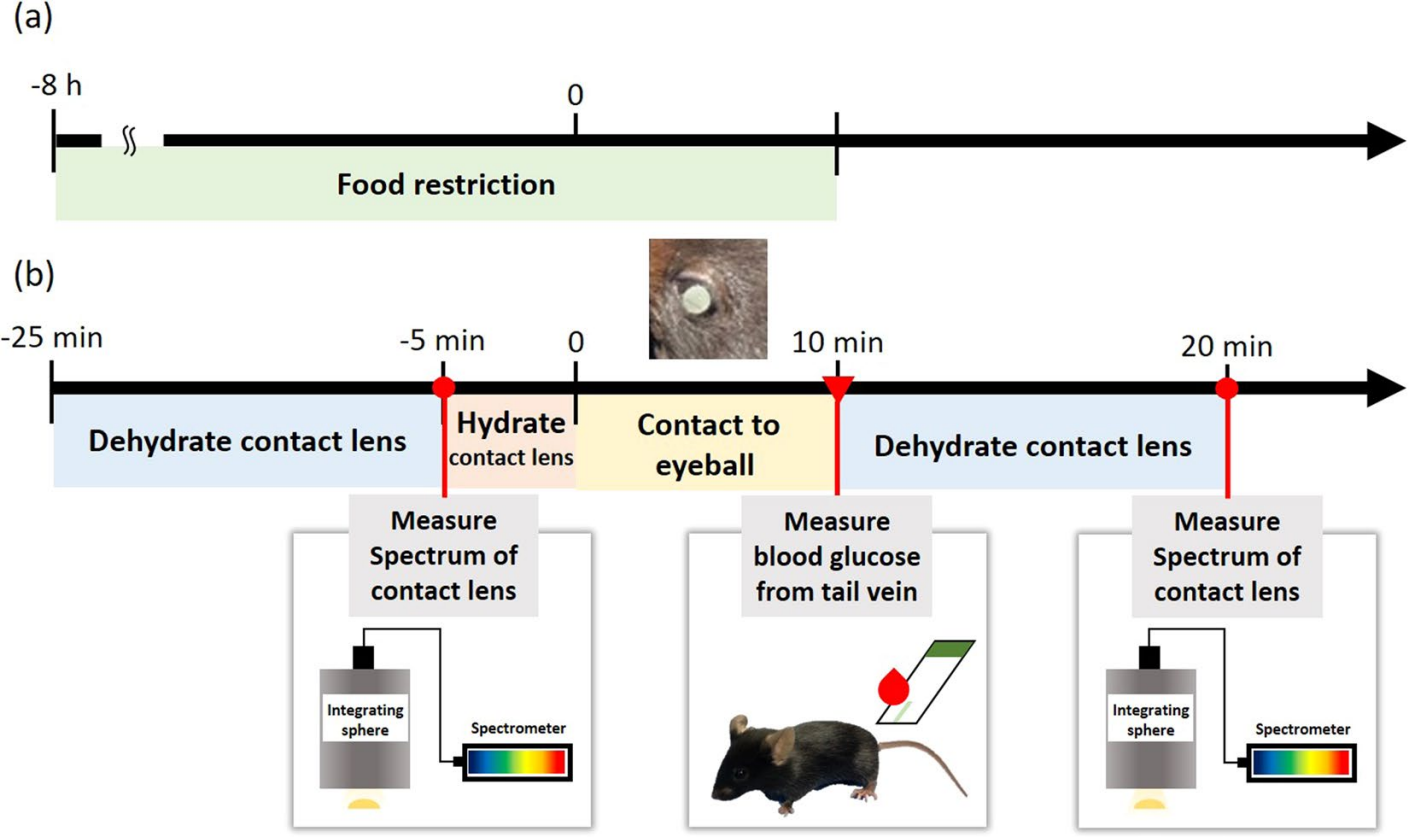
Experimental timeline schematic for animal experiments. (**a**) Timeline of inducing fasting-state for animal experiment. (**b**) Timeline of tear glucose sensing contact lens preparation, dehydration, measurement and study procedures in animal experiments.

**Figure 6:**
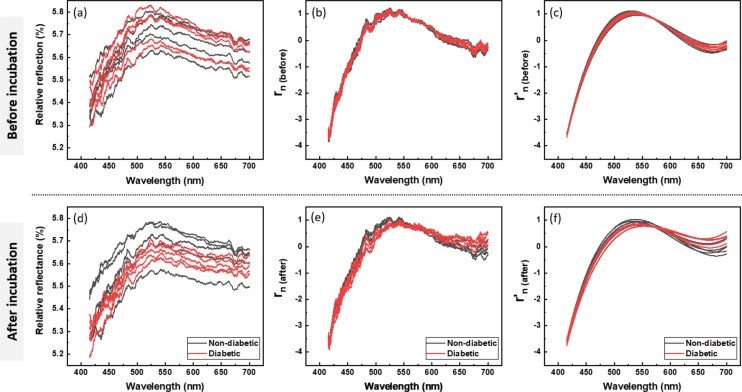
Spectrum analysis process in mice experiment. To remove the baseline difference in relative reflectance, SNV normalization was applied maintaining the original shape of contact lens reflectance. The normalized relative reflectance was 3^rd^ curve fitted and visualized. (n = 5).

The overall process is described in Fig. 5. Prior to experiments, all animals were food-restricted for 8 hours (Fig. 5(a)) and glucose were measured at the fasting state, but water was allowed. Mice were fixed using a cylindrical fixation device without anesthesia. To maintain the enzymatic activity, NECLs were immersed in deionized water and stored in a refrigerator. Before contacting mice eyeball, NECL was dried for 30 minutes and measured reflectance spectrum. And NECL was wetted in deionized water and attached in a soft state. NECL was contacted for 10 minutes and then the blood glucose was measured from the tail vein using a glucometer and a lancet, Accu-Chek Performa (Roche Diagnostics, Penzberg, Germany). For 20 minutes NECL was dehydrated on a kimwipe. The reflectance of NECL was measured in a completely dry state. The measured and processed relative reflectance were represented in Fig. 6. All procedures for the animal experiment were performed at room temperature.

**Figure 7:**
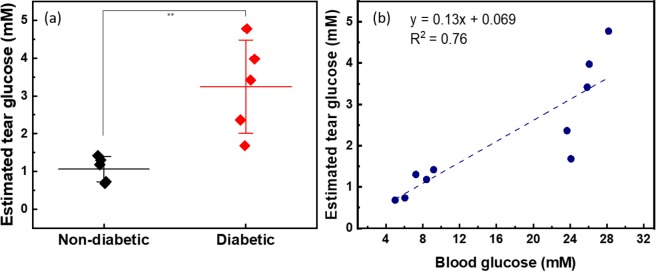
Results of tear and blood glucose measurement using mice model: (**a**) Average and STD values of tear glucose concentration of both groups. (**b**) The correlation of tear and blood glucose levels for all animals fitted to least-squares linear regression. (y = 0.12x + 0.11, R^2^ = 0.76).

NECLs were found to be stable at least a month as the structure was maintained and no release of the nanoparticles were detected with UV-VIS spectrum analysis of the buffer solution (0.9% NaCl). In addition, NECLs did not induce any sign of toxicity to the HUVECs (human umbilical vein endothelial cells) with a cell viability assay. Furthermore, the feasibility of this method using NECL glucose biosensor and spectrum analysis was evaluated by conducting *in vivo* animal experiment. The tear glucose concentrations were calculated using the calibration model established by a linear regression approach. The estimated tear glucose concentrations were plotted in Fig. 7(a). The mean are represented and error bars are a standard deviation. Figure 7(b) shows a linear relation between blood and estimated tear glucose concentrations, plotting the blood and estimated tear glucose concentrations obtained from a total of 10 mice tested in this work. The linear correlation with a statistical significance was obtained (R-square = 0.76).

Table 1 contains the blood glucose concentration and estimated tear glucose concentration, the additional information about the mice model used in this study. The diabetic mice model include type 2 diabetes and severe obesity. The body weight can be a significant indicator in the obese diabetic model, so that all mice were weighted before the day of the *in vivo* animal experiment in a non-fasting state. The actual blood glucose was measured from the blood using a glucometer approved for clinical use.

**Table 1:** Information of normal and diabetic mice model.

	Non-diabetic	Diabetic
Number of animals	3 Male, 2 Female	3 Male, 2 Female
Strain	C57BL/6	BKS.Cg-m +/Lepr/J_CRJ
Weight (g)	22.0 ± 2.1	44.6 ± 2.0
Blood glucose (mM)	7.18 ± 1.52	25.6 ± 1.61
Tear glucose (mM)	1.05 ± 0.36	3.13 ± 0.99

## Discussion

There were many trials to detect tear glucose level noninvasively or less invasively with contact lens type glucose sensor to replace invasive blood glucose measurement. However, smart contact lens with a tear glucose sensor contains an embedded sensor chip, an antenna for wireless communication, and a battery which requires sophisticated technology with higher cost^30^. Our NECL device utilizes the changes in reflection spectrum in the visible light regime with a simple, low-cost white light source, a halogen lamp. Using our reflectance spectroscopy-based approach we distinguished glucose solutions with 0.6 mM interval. Thus, our contact lens sensor provides a simple, low-cost means for tear glucose monitoring. Furthermore, NECL’s current precision could be improved with further optimization. This method may be beneficial to the patient who needs to monitor blood glucose multiple times a day but are reluctant to finger pricking.

For *in vivo* study, in the current study, we performed a tear glucose measurement with diabetic mouse model for the first time. NECLs with a diameter of ~2 mm were used to attach all areas to the mouse eyeball. Four groups of known glucose solutions (5 µL) were tested with a modified NECL. We found the most sensitive area in the entire visible spectrum from the measured reflection spectrum in the area of blue near 500 nm, the complementary color of yellow. We were able to acquire a linear regression equation and establish a calibration model from the reflectance spectrum (R^2^ = 0.87). Based on this model, tear glucose concentration in mice model were calculated and correlated with blood glucose concentration (R^2^ = 0.76). Normal and diabetic groups showed distinct glucose levels when tear and blood glucose levels were measured in a mouse model.

However, due to the high blood glucose levels in the severe diabetic mouse model induced by mutation, there could be some limitations for low glucose level measurement with animal models (Table 1). In this sense, our approach could be more suitable for healthcare purposes, especially for severe diabetic patients that demand to monitor their glucose level several times a day. Moreover, NECL’s materials have lower transmittance and can block the eyesight. Additionally, current NECLs are not considered to be reusable as the chemical reaction of cerium oxide is not reversible. For this reason, the development of locally embedding nanoparticles avoiding the pupil area is necessary to facilitate more realistic clinical application. For example, the contact lens can be manufactured in the shape of a donut with a clear central field of view. Thus, patients will be able to carry on their daily lives for 10 minutes while wearing the contact lens.

We showed a high tear glucose level in the diabetic mouse group compared to the non-diabetic mouse group using our optical system. Our research suggests that this approach may be applicable for monitoring of tear glucose level. Our non-invasive monitoring approach may offer another option that would be an alternative to current invasive glucose monitoring techniques for diabetic patients.

## Methods

### Nanoparticle embedded contact lens fabrication

NECL were obtained from Hanyang University (Seoul, South Korea) on the day that NECLs were fabricated. NECLs were embedded with CNP-PEG-GOX nanoparticles. NECLs were transported to our laboratory immersed in distilled water in a glass tube and packed in a box filled with ice within 24 hours. Right after NECLs arrived in the experiment room, which were stored in the refrigerator. All NECLs were used within three days of arrival. Before the experiment, NECLs were cut to 2 mm to adapt to a size suitable for the cornea of experimental mice.

### Calibration

Lybliss 0.15% Eye Drops (single use, 1 mL) were purchased from Huons Medicare. D-(+)-glucose were purchased from Sigma-Aldrich. The glucose solution was prepared by dissolving D-(+)-Glucose (Sigma-Aldrich, USA) at the concentrations of 0, 0.6, 1.2, 2.4 mM in artificial tear fluid, Lybliss 0.15% Sodium hyaluronate Eye Drops, single use, 1 mL (Huons Medicare, Busan, South Korea).

### Animal care

We used 10 to 12 weeks old mice, 3 males and 2 females from each strain (BKS.Cg-m+/Lepr/J_CRJ, C57BL/6), for *in vivo* glucose sensing. All mice were housed in the Laboratory Animal Resource Center at the Gwangju Institute of Science and Technology and maintained under a 12 h light/dark cycle and fed with granulated food and water *ad libitum*. All animal procedures and care were approved by the Institutional Animal Care and Use Committee board (IACUC) of the Gwangju Institute of Science and Technology and all experiments were performed in accordance with relevant guidelines and regulations.
